# Endothelial Cells, Ankaferd Hemostat, and Estradiol

**DOI:** 10.4274/tjh.2015.0143

**Published:** 2016-08-19

**Authors:** Yasemin Ardıçoğlu, Nejat Akar, İbrahim Haznedaroğlu

**Affiliations:** 1 TOBB-ETU Hospital, Ankara, Turkey; 2 Hacettepe University Faculty of Medicine, Department of Hematology, Ankara, Turkey

**Keywords:** endothelium, Ankaferd, Estradiol

We previously demonstrated the effects of Ankaferd hemostat (AH) on human umbilical vein endothelial cells (HUVECs) in Turkish Journal of Hematology [1]. Endothelial cells adhered to each other within seconds and critical intracellular transcription factors were activated just after the application of AH (5 µL) to the HUVECs (Figure 1A). Rapid vital endothelial cell adherence/aggregation is reversible and could be reversed within 24 h (Figure 1B).

We further determined that the cellular effects of AH on HUVECs are clinically important in pharmacobiological hemostasis [1,2,3]. Endothelial cells are involved in a range of pathophysiological processes including hemostasis, inflammation, and angiogenesis [4], all of which are directly related to the effects of AH [1,2,3]. However, the relevant receptors on the surface of HUVECs and the molecules inside the content of AH affecting the endothelial cells remain unknown. Since HUVECs express estrogen receptor (ER) beta [4] and rapid HUVEC cellular responses to estrogen can be mediated by estrogen binding to ER [5], we herein aimed to investigate the estradiol content of AH. Estradiol concentration is found to be very high in AH (1452.6 pg/mL), whereas progesterone level is 6.06 ng/mL. Those results suggest novel hypotheses that shall be tested in future investigations regarding the interrelationships of vascular endothelial cells, hemostasis, and estradiol inside AH.

## Figures and Tables

**Figure 1A:**
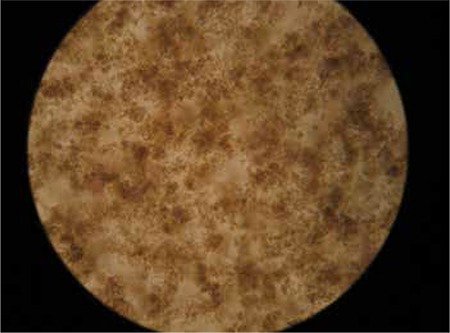
A. Human umbilical vein endothelial cells adhered to each other, within seconds, just after the application of Ankaferd hemostat (5 µL).

**Figure 1B:**
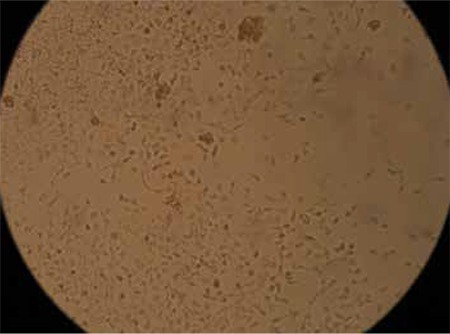
Reversible vital endothelial cell adherence/aggregation in human umbilical vein endothelial cells 24 h after application of Ankaferd hemostat (5 µL).
